# Assessment of functionality using the WHODAS 2.0 in community-dwelling elderly individuals: A scoping review

**DOI:** 10.1097/MD.0000000000043372

**Published:** 2025-07-25

**Authors:** José Felipe Costa da Silva, Luciana Araújo dos Reis, Catharinne Angélica Carvalho de Farias, Silvana Loana de Oliveira-Sousa, Felipe León Morillas, Thaiza Teixeira Xavier Nobre

**Affiliations:** a Public Health at the Federal University of Rio Grande do Norte, Natal, Rio Grande do Norte, Brazil; b Department of Health I, State University of Southwest Bahia, Jequié, Bahia, Brazil; c Faculty of Health Sciences of Trairí, Federal University of Rio Grande do Norte, Santa Cruz, Rio Grande do Norte, Brazil; d Department of Physiotherapy, Physiotherapy and Health, University of Murcia, Murcia, Spain; e Department of Physiotherapy, Catholic University of Murcia UCAM, Murcia, Spain.

**Keywords:** disability and health, elderly, elderly health, primary health care, international classification of functioning

## Abstract

In elderly individuals, it is more common to observe a decline in their functionality, resulting in difficulties in performing daily activities and participating in the community. To mitigate this decline, longitudinal monitoring of the elderly individual is necessary, along with the use of instruments such as the World Health Organization Disability Assessment Schedule 2.0 (WHODAS 2.0), which evaluates functionality across various aspects. The scope is to analyze the use of WHODAS 2.0 as an instrument for assessing functionality in community-dwelling elderly individuals. A scoping review was conducted on WHODAS 2.0 as an instrument for assessing functionality in community-dwelling elderly individuals. The descriptors “Disability Assessment Schedule II”; “WHODAS 2.0”; “WHODAS”; “Aged”; “Elderly”; “Aging”; “Primary Health Care”; “Primary Care”; “Primary Healthcare” were used in the following databases: Medline, Embase, CINAHL, Scopus, Web Of Science, Embase, Science Direct, and Google Scholar. The initial collection resulted in a total of 425 studies addressing the topic. After readings using inclusion and exclusion criteria, 14 studies were included for analysis. The majority of studies were conducted in primary healthcare, with a greater number of women in various countries. The domains that most impacted functionality were mobility, activities of daily living, and social participation. Conversely, interpersonal relationships and self-care were the least affected domains. It is notable that WHODAS 2.0 is an instrument that can be used in community-dwelling elderly individuals. The domains that most negatively influenced functionality were mobility, activities of daily living, and social participation.

## 1. Introduction

The reduction in mortality and increased life expectancy contribute to the growing number of elderly individuals.^[1,2]^ These demographic characteristics directly affect the population’s health conditions, altering the types of most common diseases, with a noticeable accelerated growth in noncommunicable chronic diseases in this population. This group of diseases increases levels of human disability and negatively affects functionality and quality of life, especially among the elderly.^[3]^

Human functionality is a comprehensive and macro term in the health and disease process, encompassing various elements of the body, its functions and structures, human activities, and the individual’s participation in social, personal, and environmental processes that can be barriers or facilitators in the individual’s life. This holistic view of the person contributes to better healthcare, especially for elderly individuals monitored by Primary Health Care (PHC).^[4,5]^

The functionality of the elderly can be assessed in various ways, with particular emphasis on the World Health Organization Disability Assessment Schedule 2.0 (WHODAS 2.0) instrument, available in versions with 12 and 36 items, which is easy to apply. It is used to assess the level of functionality across 6 life domains (cognition, mobility, self-care, social interaction, activities of daily living (ADLs), and participation in society), and can be administered through interviews, with a caregiver, or self-administered. Its scoring varies according to characteristics such as age, reduced mobility, depression, hypertension, among other issues.^[6,7]^

Interdisciplinary interventions in elderly healthcare are important for maintaining functionality, especially in elderly individuals living in the community. Many of these individuals actively participate in health promotion activities and prevention of health issues, an important strategy in public health care.^[8]^ Primarily, for effective healthcare interventions in the elderly, a thorough individual assessment is necessary. In this sense, WHODAS 2.0 seems to be an applicable instrument. Thus, the main objective of this scoping review is to analyze the use of WHODAS 2.0 as an instrument for assessing functionality in community-dwelling elderly individuals.

## 2. Objective

The main objective of this scoping review is to analyze the use of WHODAS 2.0 as an instrument for assessing functionality in community-dwelling elderly individuals.

## 3. Methodology

Protocol and registration: The research protocol was developed using the preferred reporting items for systematic reviews and meta-analysis protocols (PRISMA-P)^[9]^ and registered in the open science framework (DOI 10.17605/OSF.IO/MAQVW).

Research question: for the construction of the research question, the PCC acronym was used, which involves participants, concept, and context, as can be observed below:

Participants: individuals over 60 years old, without sex restrictions, who may be healthcare system users or community residents.

Concept: this review will consider studies that explore actions to assess the functionality of elderly individuals using WHODAS 2.0.

Context: this review will consider studies focused on elderly individuals associated with primary care or those living in the community, without limiting to any geographical region. Based on these aspects, the following research question was formulated: What are the results of primary studies using WHODAS 2.0 in the assessment of community-dwelling elderly individuals?

Types of studies: this scoping review will consider quantitative study designs with an observational approach.

Eligibility criteria: studies published from January 2012 to March 2023 were included to encompass the most recent studies, in English, Portuguese, or Spanish languages, with quantitative and observational study designs, including elderly individuals (aged 60 or over), who were evaluated regarding their functionality using WHODAS 2.0, in PHC or elderly individuals living in the community. Qualitative investigations, theoretical essays, research protocols, methodological articles, theses and dissertations, editorials, letters to the editor, abstracts, expert opinions, correspondences, and book chapters were excluded.

Sources of information: primary research studies published in scientific journals were included in this study. Therefore, the following electronic databases were consulted: Medical Literature Analysis and Retrieval System Online (Medline), Scopus, Google Scholar, Web of Science, Embase, CINAHL, ScienceDirect.

Search strategies: the search included the following terms included in the medical subject headings (MeSH): World Health Organization “disability assessment schedule II”; “WHODAS 2.0”; “WHODAS”; “Aged”; “Elderly”; “Aging”; “PHC”; “Primary Care”; “Primary Healthcare.” Boolean terms “AND” and “OR” were used to construct search strategies adapted to each database. An example of the results of a preliminary search is included in Table 1.

**Table 1:** Preliminary research with search strategies and filters.

Strings de Busca	Records	Database	Filters
(“World Health Organization Disability Assessment Schedule II” OR “WHODAS 2.0” OR WHODAS) AND (Aged OR Elderly OR Aging) AND (“Primary Health Care” OR “Primary Care” OR “Primary Healthcare”)	39	Medline	Languages: English, Spanish and Portuguese
	43	SCOPUS	
	47	Google scholar	
	39	WEB OF SCIENCE	
	55	EMBASE	Publication period: 2012 to 2023
	10	CINAHL	
	192	Science Direct	
	425	All bases	

WHODAS = World Health Organization Disability Assessment Schedule.

Source: Own authorship, 2023.

Study selection: ffter the bibliographic search, all identified records were exported from the databases in EndNote, Export, Refman/RIS, or text formats and included in the Rayyan QCRI platform. This platform, developed by the Qatar Computing Research Institute and available online for free, offers a diverse range of resources, including uploading citations in various formats, navigation, and automatic extraction to exclude duplicate citations. For this study, Rayyan QCRI was used for duplicate exclusion and was necessary for title and abstract analysis.^[10]^

The full text of selected citations was evaluated in detail according to the inclusion criteria. The reasons for excluding full-text articles that did not meet the inclusion criteria were recorded and reported in the review. The research results were presented in full in the PRISMA flow diagram.

Data extraction: after removing duplicate publications, study selection was initiated by 2 independent researchers, starting with reading titles and subsequently abstracts based on inclusion and exclusion criteria. Selected studies were read in full, and additional exclusions were made after complete study reading. For data extraction, an instrument developed by the authors in spreadsheet format was used, containing information about Author, Year, Journal, Study Design, Sample, Age, Sex, Objective, Assessment Scales, Worst WHODAS 2.0 Scores, Highest Score (Worst), Lowest Score (Best), and Conclusion.

The data extraction tool was modified and reviewed as necessary during the data extraction process for each included article. Data analysis and presentation: the data were descriptively mapped and presented through tables, charts, and figures showing the distribution of articles by publication period, country, and main variables found. A narrative summary of all results is included in the tables and figures, describing the main findings. Ethics and disclosure: all data in this study are from published studies, and therefore, no ethics committee approval is required since no patient recruitment is necessary, and no personal privacy is involved.

## 4. Results

**Figure 1.**
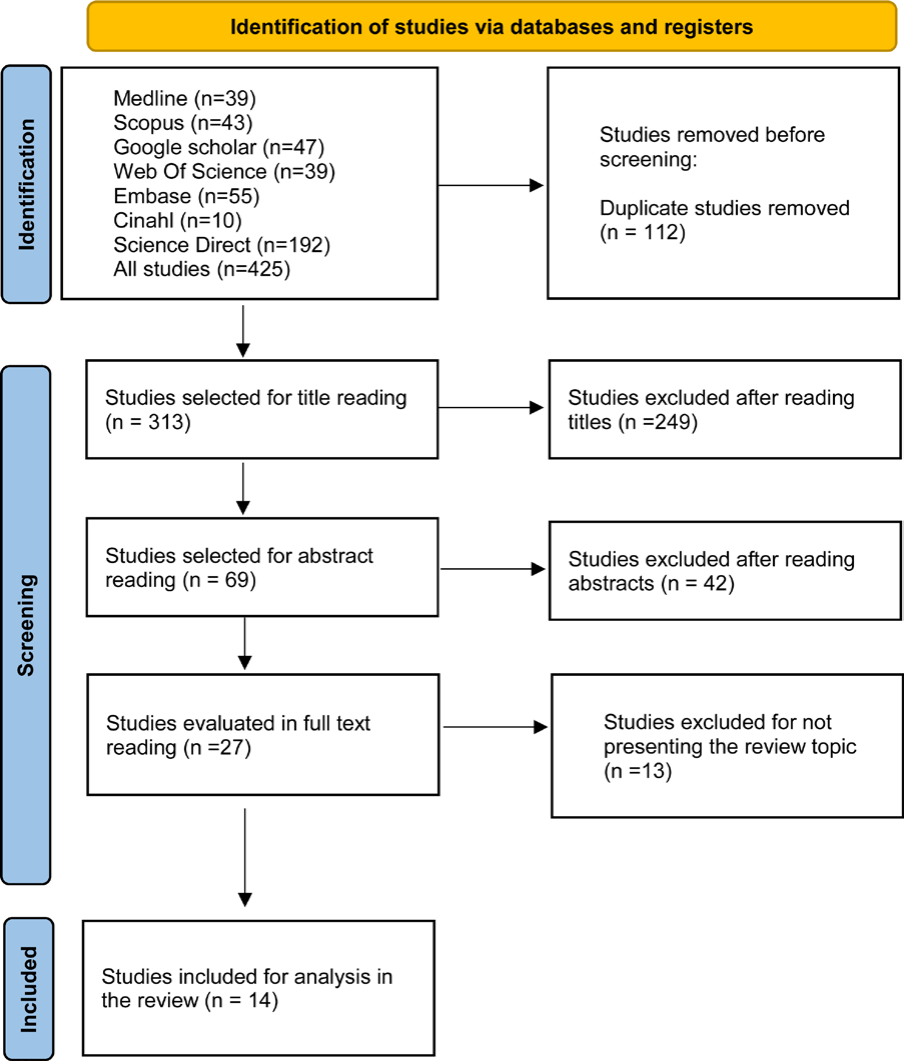
Diagram of the study selection process, 2023. Cinahl = Cumulative Index to Nursing and Allied Health Literature, Embase = Excerpta Medica database, Medline = Medical Literature Analysis and Retrievel System Online. Source: study data.

The database search resulted in 425 studies. After removing duplicates, 313 records remained for title and abstract review. Twenty-seven studies were retained for full-text review, and 14 studies were included in data extraction and literature synthesis. The results of the study identification and selection phases were described in detail based on the Preferred Reporting Items for Systematic Reviews and Meta-Analyses (PRISMA),^[11]^ as observed in Figure 1.

Table 2 presents the main characteristics of the studies included in this review, including the profile of the elderly, totaling 20,667 elderly individuals in this scoping review. The most frequent years were from 2019 onwards, with researchers from Brazil and Portugal. All studies had a cross-sectional design, and the number of elderly individuals included ranged from 62 to 12,126. There was a prevalence of females in most samples. Further information can be observed in Table 2.

**Table 2:** Characterization of studies included in the review, 2023.

Study	Country	Magazine	Sample	Average age (SD) yr/age – n – %	Sex, n (%)	
					M	F
Almazán-Isla et al, 2014^[12]^	Spain	Disability and Health Journal	1216	69.4 (11.3)	566 (46.6)	650 (53.5)
Ćwirlej-Sozańska et al, 2020^[13]^	Poland	Annals of Agricultural and Environmental Medicine	498	82.66 (3.44)	152 (30.52)	346 (69.48)
Dernek et al, 2015^[14]^	Turkia	International Journal of Rehabilitation Research	200	72.3 (5.3)	107 (53.5)	93 (46.5)
Duba et al, 2012^[15]^	India	International Psychogeriatrics	1000	72.53 (5.87)	454 (45.4)	546 (54.6)
Grassi et al, 2020^[16]^	Europe, Germany, Italy, England, Spain, Switzerland and Israel	Health and Quality of Life Outcomes	3142	73.7 (5.6)	1550 (49.3)	1592 (50.7)
Huang et al, 2015^[17]^	Taiwan	Disability and Rehabilitation	12 126	65 and 74 – 2308 – 19.03%	4514 (37.5)	7612 (62.5)
				75 and 84 – 5827 – 48.05%		
				≥ 85 – 3991 – 32.91%		
Mournet et al, 2019^[18]^	USA	Clinical Gerontologist	62	72 (9.07)	20 (32)	42 (68)
Oliveira et al, 2019^[19]^	Brazil	Apunts Sports Medicine	654	60 and 69–59.2%	288 (44)	366 (56)
Park et al, 2021^[20]^	Republic of Korea	BMC Geriatrics	885	70.3 (6.2)	231 (32.9)	594 (67.1)
Rocha et al, 2021^[21]^	Brazil	UNIPAR Health Sciences Archives	129	≤ 79 yr old – 104–80.6%	32 (24.8)	97 (75.2)
				≥ 80 yr old – 25–19.4%		
Silva et al, 2014^[22]^	Portugal	Disability and Health Journal	251	70.87 (7.76)	55 (21.9)	196 (78.1)
Silva et al, 2015^[23]^	Portugal	Physics therapy	504	70.9 (7.5)	338 (67.1)	166 (32.9)
Silva et al, 2022^[24]^	Brazil	Health and Research	100	60 to 69–52 – 52%	19 (19)	81 (81)
				70 to 79–34 – 34%		
				≥80 – 14 – 14%		
Watfe et al, 2020^[25]^	Brazil	Journal Life	1375	60–69 750–54.6%	556 (40.5)	819 (59.5)
				70–79 437–31.8%		
				≥80 – 188–13.6%		

F = female, M = male, SD = standard deviation.

Source: Studies included, 2023.

The results of these cross-sectional studies provide a comprehensive overview of demographic and health characteristics in elderly populations around the world. In Spain, Almazán-Isla et al (2014)^[12]^ observed a sample of 1216 participants with a mean age of 69.4 (±11.3) years, with a prevalence of women (53.5%). In Poland, Ćwirlej-Sozańska et al (2020)^[13]^ analyzed 498 individuals with a notably high mean age of 82.66 (±3.44) years, with 69.48% being women.

Other noteworthy results include studies by Grassi et al (2020),^[16]^ who investigated a broad sample in various European countries, finding a mean age of 73.7 ± 5.6 years and an almost equitable gender distribution. Additionally, Huang et al (2015) presented data from Taiwan, highlighting a significant sample of 12,126 participants across age groups, with 62.5% being women. Studies conducted in the United States,^[18]^ Brazil,^[19,23,25]^ Portugal,^[22,23]^ and Taiwan^[17]^ collectively contribute to understanding the demographic and health characteristics of the elderly population in different contexts and health conditions.

Table 3 presents the methodology and main results of the studies. Although the objective of the present study was to evaluate the use of WHODAS 2.0, some studies associated it with other instruments and assessment scales. Overall, the profile or condition in which elderly individuals were found was mostly associated with APS, or conditions such as dementia, rural elderly, or low income. The domains that most affected functionality were mobility, ADLs, and community participation.

**Table 3:** Methodology and main results of the included studies, 2023.

Study	Objective	Assessment instruments	Health condition/group	Conclusion	Worst scores in WHODAS 2.0
Almazán-Isla et al, 2014^[12]^	Determine and characterize prevalence of disability among the rural middle-aged and elderly population of the district Spanish from Cinco Villas.	WHODAS 2.0	Rural area residents	Disability was detected by the WHODAS global score in 604 of a total of 1214 people, that is, a prevalence of 49.8% CI 95% (46.9 and 52.5), with corresponding values for mild, moderate, severe and extreme, being 26.8%, 16.0%, 7.6% and 0.1%, respectively. Disability was more common among individuals diagnosed with dementia, chronic liver disease, severe mental illness and stroke.	Life activities; mobility; social participation
Ćwirlej-Sozańska et al, 2020^[13]^	To assess the level of disability and quality of life in a population of men and women aged 80 and over living in southeastern Poland.	WHODAS 2.0/WHOQOL-BREF	Long-lived 80 yr or more	The average general level of disability was 37.41, with women having a higher level of general disability than men (38.94 vs 33.94).	Life activities; mobility; social participation
Dernek et al, 2015^[14]^	Investigate disability in community-dwelling individuals aged 65 and over using the ICF and WHODAS 2.0 checklist	WHODAS 2.0/ICF	APS seniors	Both the ICF checklist and WHODAS 2.0 identified a considerable proportion of elderly people with a lifetime disability. However, the proportions of people with disabilities showed a statistically significant difference in the domains of the 2 instruments relevant to mobility, self-care and participation in society.	Life activities; mobility; social participation
Duba et al, 2012^[15]^	To simultaneously examine the biomedical and social determinants of disability among the elderly population in a rural community in southern India.	WHODAS 2.0/CSID/GMS/CERAD	Rural community	Old age, illiteracy, hunger, poor nutrition, arthritis, hearing impairment, gastrointestinal and respiratory diseases, dementia and travel costs to health facilities have significantly increased the risk of disability, hypertension, diabetes and depression.	Mobility; life activities; social participation
Grassi et al, 2020^[16]^	To explore the characteristics of quality of life and level of functionality among elderly people in European countries and the association of mental disorders.	WHODAS 2.0 (12 items)/WHOQOL-bref	Rural and urban community	The majority of subjects reported good levels of HRQoL (56.6%) and self-rated health (62%), with no or mild disability (58.8%). There was a linear decrease in HRQoL and level of functionality with increasing age. Elderly people with ICD-10 mental disorders had a worse quality of life and lower level of functionality.	Mobility; self-care, social participation
Huang et al, 2015^[17]^	To analyze the disability status of Taiwanese elderly people with dementia using the WHODAS 2.0	WHODAS 2.0	Insanity	WHODAS 2.0 scores showed that patients with dementia had global activity limitation and restriction of participation in all domains. Dementia-induced disability was prominent in male patients across all WHODAS 2.0 domains.	Life activity; social participation, cognition
Mournet et al, 2019^[18]^	Examine associations between domains of functional impairment and 2 forms of social disconnection that are empirically linked to suicide later in life – low (or frustrated) belonging and perceived burden on others	WHODAS 2.0/INQ-R/QIDSC	APS seniors	Greater perceived overload was associated with greater impairment in activities of daily living, while greater frustrated belonging was associated with greater impairment in social functioning, when controlling depressive symptoms and age.	Social participation; mobility; cognition
Oliveira et al, 2019^[19]^	Analyze the relationship between the practice of activity physical and functionality of the elderly, mediated by behavior sedentary.	WHODAS 2.0/ IPAQ	APS seniors	The relationship between intense physical activity and the functionality of elderly people in PHC is weak, as is the fact that sedentary behavior reduces the effect of vigorous activities on the functionality of these elderly people.	*
Park et al, 2021^[20]^	To investigate the prevalence of osteosarcopenia in the community over 60 years of age and assess whether osteosarcopenia is associated with disability, frailty and depression.	WHODAS 2.0 (12 items)/The Kaigo-Yobo checklist (frailty)/GDSSF-K	APS seniors	Osteosarcopenia is a relatively common group of diseases in the elderly community that can cause deterioration in health outcomes.	*
Rocha et al, 2021^[21]^	Assess the functional capacity of elderly people according to WHODAS 2.0	WHODAS 2.0/IPAQ	APS seniors	For the majority of elderly people interviewed, there was changes in their functionality, with a higher level of disability in the domains of participation in society, cognition and mobility, and the general classification ranged from mild to moderate.	Participation in society; cognition; mobility
Silva et al, 2014^[22]^	To determine whether personal factors, pain, depression and physical activity are associated with self-reported and performance-based disability in elderly people aged ≥ 60 years treated at UBS	WHODAS 2.0 (12 items)/SPPB	APS seniors	The results indicate that primary health care interventions should target pain intensity, depressive symptoms, and physical activity as a way to prevent or decrease self-reported performance-based disability.	Life activities; mobility; social participation
Silva et al, 2015^[23]^	To investigate the association between the World Health Organization disability assessment scheme 2.0 (WHODAS 2.0) and the short physical performance battery (SPPB) for older adults in primary care	WHODAS 2.0 (12 items)/SPPB	APS seniors	Regression analysis showed that the SPPB total score explained 41.7% of the variance in WHODAS 2.0 scores. The results of this study confirm that self-report and performance-based measures are related to different aspects of functioning	Life activities; mobility; social participation
Watfe et al, 2020^[25]^	Investigate the prevalence and factors associated with disabilities and areas of lives affected by disabilities in elderly people registered at UBS affiliated with the ESF in 2 large Brazilian cities: São Paulo and Manaus	WHODAS 2.0 (12 items)	Low income communities	The prevalence of global disability was higher in Manaus (66.2% vs 56.4% in São Paulo). The number of consultations with a family doctor was not associated with disability. The high prevalence of disability and associated risk factors indicates that public primary healthcare is not meeting the needs of the elderly in both cities.	Life activities; mobility; social participation
Silva et al, 2022^[24]^	Verify the relationship between lifestyle habits, life purpose and functionality of elderly people living in a community center	WHODAS 2.0 (12 items)/Ryff and Keyes Scale	Community centers	It was noted that members of the community center, practitioners of educational and cultural activities, showed better overall functionality (*P* = .03) and in the domains “Participation in society” (*P* = .00) and “Life activities” (*P* = .01)	*

APS = Primary Health Care (Portuguese abbreviation), CERAD = consortium to establish a registry for Alzheimer Disease, CSID = community screening instrument for dementia, ESF = Family Health Strategy, GDSSF-K = geriatric depression scale-short form-Korean, GMS = geriatric mental state, HRQOL = health-related quality of life, ICF = International Classification of Functioning, Disability, and Health, INQ-R = interpersonal needs questionnaire –revised, IPAQ = International physical activity questionnaire, QIDS = quick inventory of depressive symptomatology, QIDSC = Questionnaire for the Inventory of Depression and Suicide Cognitions, SPPB = short physical performance battery, UBS = Basic Health Unit, WHODAS 2.0 = World Health Organization Disability Assessment Schedule 2.0, WHOQOL-Bref = World Health Organization Quality of Life – Brief Version.

Source: Studies included, 2023.

* Data not available.

Based on the results presented in Table 4 for the domains of WHODAS 2.0, which assess functionality and disability in different areas of life, it is possible to identify performance patterns. In the Mobility domain, 5 studies^[12,13,15,16,23]^ indicated poorer functionality in these groups of elderly individuals. Similarly, in the domain of ADLs and social participation, 3 studies^[18,21,25]^ considered the areas where the elderly have the most difficulty achieving, whereas interpersonal relationships and self-care are the least affected domains.

**Table 4:** Representation of the most and least affected domains according to WHODAS 2.0.

Study	Domains most affected in functionality			Domains least affected in functionality		
	Mobility	Life activities	Social participation	Self-care	Interpersonal relationships	Social participation
Almazán-Isla et al, 2014^[12]^	–	–	–	–	–	–
Ćwirlej-Sozańska et al, 2020^[13]^	–	–	–	–	–	–
Dernek et al, 2015^[14]^	–	–	–	–	–	–
Duba et al, 2012^[15]^	–	–	–	–	–	–
Grassi et al, 2020^[16]^	–	–	–	–	–	–
Huang et al, 2015^[17]^	–	–	–	–	–	–
Mournet et al, 2019^[18]^	–	–	–	–	–	–
Oliveira et al, 2019^[19]^	*	–	–	–	–	–
Park et al, 2021^[20]^	*	–	–	–	–	–
Rocha et al, 2021^[21]^						
Silva et al, 2014^[22]^						
Silva et al, 2015^[23]^						
Watfe et al, 2020^[25]^						
Silva et al, 2022^[24]^	*					

WHODAS 2.0 = World Health Organization Disability Assessment Schedule 2.0.

Source: studies included, 2023.

* Data not available.

## 5. Discussion

Primary Health Care is an organizational structure within healthcare systems aimed at addressing the most common problems faced by community members, including a growing segment in recent years, the elderly. Among the various services offered to this population, care should be based on multidimensional assessment, education, and family support, primarily focusing on preventing health issues and promoting well-being.^[26,27]^

This level of healthcare plays a fundamental role in addressing the needs of the elderly, aiming to manage their care, develop care plans, and, above all, conduct a multidimensional assessment to prevent and treat common age-related conditions. Among its many responsibilities are fostering supported self-care, health monitoring, promoting health education, and offering family support.^[28]^

Indeed, most of the studies included here focused on elderly individuals who were part of PHC. This setting hosts various collective and individual activities often geared towards older adults. These actions and services were mentioned in de Castro et al study (2018),^[5]^ which observed group activities, meetings, discussions, lectures, and guidance sessions in waiting rooms, as well as individual guidance during multiprofessional consultations and referrals to specialized services.

The average age found in the studies included here was around 70 years old. This average has been increasing in studies involving community-dwelling elderly individuals, reflecting a widely observed demographic transition worldwide. Older adults aged 75 and above, often considered the most long-lived, were highlighted in the participant samples. Observational studies have found a correlation between decreased performance in ADLs and this age group.^[29,30]^

The study objectives cover a wide range of health and functionality issues across different population groups. They reveal a prevalence of disabilities in specific populations, such as elderly individuals in rural areas of Spain, individuals aged 80 or older in southeastern Poland, and elderly residents in rural communities in India. Beyond age-related decline in function, the cognitive state without alterations contributes to preventing health issues when combined with supervised exercises.^[31]^

Elderly individuals residing in rural regions of various countries appear to have less access to healthcare services. This was observed in the study in India, which noted considerable rural-urban inequality that disadvantages rural residents, with healthcare utilization rates being 7 percentage points higher among urban elderly residents than their rural counterparts. Similarly, Chinese elderly individuals engaged in agriculture, with low incomes, solitary lives, and depression, had higher predicted needs for community services.^[32,33]^

The WHODAS 2.0 is commonly used to assess community-dwelling elderly individuals, often in conjunction with other instruments depending on the study’s objectives. It can be applied in 12, 24, and 36-question formats. Five studies were found to use the 12-question version, offering advantages such as reduced administration time. Despite its lower number of questions, it has equal validity in clinical and healthcare research, showing strong correlations with various other measures of activity limitations.^[34,35]^

Moreover, several studies explore associations between biomedical, social, and behavioral factors and the presence of disabilities in the elderly. For instance, the study in India utilized the WHODAS 2.0 and the community screening instrument for dementia among other instruments. These investigations aim to understand the complex interactions between health conditions, cognitive and social functioning. Among these variables, it is evident that human gait, cognition, and falls are closely related to elderly functional health, especially in those with some degree of dementia.^[32]^

The research results provide a comprehensive overview of the prevalence and determinants of disability across various domains. An analysis using the WHODAS 2.0 revealed a significant prevalence of disability in the elderly population. The highest disability scores are concentrated in areas such as mobility, ADLs, and social participation.^[36]^

The mobility of elderly individuals warrants greater attention from healthcare professionals and families. Reduced movement in daily living and social spaces leads to a series of physical, social, and mental health modifications. Some more complex comorbidities, such as loss of muscle mass and sarcopenia, result in decreased mobility, increased falls, recurrent hospitalizations, and higher mortality rates.^[37]^

Longitudinal studies in the elderly can highlight characteristics of this more fragile population. For example, a study in Costa Rica and the United States found that the risk of mortality significantly increases among frail elderly individuals, particularly at advanced ages, more prevalent in males and smokers.^[38]^

Studies have shown that ADLs and instrumental ADLs also directly influence the good functionality of elderly individuals. Cohort studies support these findings by observing that community-dwelling elderly individuals who do not engage in ADLs and recreational activities or perceive life motivations are significantly associated with an increased risk of mortality. Another study that evaluated 31,464 elderly individuals in India found that 3% reported severe disability in ADLs and 6% reported severe disability in instrumental ADLs, with those not involved in any physical activity more likely to report severe ADLs.^[39,40]^

Regarding domains with less impact on the functional health of elderly individuals, it is possible to observe that in the Interpersonal Relationships domain, excellent functionality was found. This indicates that participants have healthy and satisfactory relationships, including family, neighborhood, and group relationships.^[41]^

Indeed, a study involving 2839 60-year-olds demonstrated that both neighborhood environment and social participation had significant positive effects on elderly health across all samples. Another study noted that social isolation is a significant and potentially modifiable risk affecting a significant proportion of the elderly population, with the most common profile being unmarried, male, with low education levels, and low income.^[41,42]^

The Self-care domain also deserves attention. It involves aspects such as feeding, dressing, and personal hygiene, and presented lower scores in 7 studies, also being one of the best domains in the findings. Overall, studies addressing elderly self-care often relate to diseases such as diabetes. Supporting these findings, a study on elderly individuals living with diabetes in Primary Health Units in Fortaleza/Ceará, northeast Brazil, found that healthy eating, dietary guidance, and foot examinations were the main aspects of self-care.^[43]^

This review has some limitations. Only cross-sectional studies were included in the methodology, and the risk of bias, evidence quality, and strength of recommendations were not evaluated. Another limitation was the exclusion of studies from gray literature. Future studies should include other study designs and different functional assessment instruments for comparison with the WHODAS 2.0.

## 6. Conclusion

The WHODAS 2.0 is a functional assessment instrument that can be used in community-dwelling elderly individuals. The results of this review identified studies that acknowledge this usage, and the domains of the WHODAS 2.0 reveal aspects that healthcare professionals should pay more attention to, particularly structures and bodily functions associated with mobility, ADLs, and elderly community participation.

Effective strategies from primary healthcare teams are needed to address not only physical and biological aspects but also the involvement of elderly individuals in the community, given that this domain is one of the highlights that can reduce functionality and consequently the quality of life of community-dwelling elderly individuals.

## Author contributions

**Data curation:** Felipe León Morillas.

**Formal analysis:** Felipe León Morillas.

**Investigation:** José Felipe Costa da Silva.

**Methodology:** José Felipe Costa da Silva, Thaiza Teixeira Xavier Nobre.

**Project administration:** Silvana Loana de Oliveira-Sousa.

**Supervision:** Catharinne Angélica Carvalho de Farias.

**Validation:** Silvana Loana de Oliveira-Sousa.

**Visualization:** Silvana Loana de Oliveira-Sousa, Thaiza Teixeira Xavier Nobre.

**Writing – original draft:** Luciana Araújo dos Reis.

**Writing – review & editing:** Catharinne Angélica Carvalho de Farias, Thaiza Teixeira Xavier Nobre.
